# The Relevance of the Colon to Zinc Nutrition

**DOI:** 10.3390/nu7010572

**Published:** 2015-01-14

**Authors:** Geetha Lavaniya Gopalsamy, David H Alpers, Henry J Binder, Cuong D Tran, B S Ramakrishna, Ian Brown, Mark Manary, Elissa Mortimer, Graeme P Young

**Affiliations:** 1 School of Medicine, Flinders University of South Australia, Adelaide 5001, Australia; E-Mails: mrbmrsb1@gmail.com (I.B.); elissa.mortimer@flinders.edu.au (E.M.); graeme.young@flinders.edu.au (G.Y.); 2 CSIRO Food and Nutrition Flagship, Gate 13, Kintore Ave, Adelaide, SA 5000, Australia; E-Mail: cuong.tran@csiro.au; 3 School of Medicine, Washington University, St Louis, MO 63110, USA; E-Mails: DAlpers@dom.wustl.edu (D.H.A.); manary@kids.wustl.edu (M.M.); 4 School of Medicine, Yale University, New Haven, CT 06520, USA; E-Mail: henry.binder@yale.edu; 5 School of Medical Sciences, Faculty of Health Sciences, The University of Adelaide, SA 5005, Australia; 6 SRM Institutes for Medical Science, 1 Jawaharlal Nehru Road, Vadapalani, Chennai 600 026, India; E-Mail: drramakrishna.bs@simshospitals.com

**Keywords:** colon, zinc, mineral absorption, fermentation

## Abstract

Globally, zinc deficiency is widespread, despite decades of research highlighting its negative effects on health, and in particular upon child health in low-income countries. Apart from inadequate dietary intake of bioavailable zinc, other significant contributors to zinc deficiency include the excessive intestinal loss of endogenously secreted zinc and impairment in small intestinal absorptive function. Such changes are likely to occur in children suffering from environmental (or tropical) enteropathy (EE)—an almost universal condition among inhabitants of developing countries characterized by morphologic and functional changes in the small intestine. Changes to the proximal gut in environmental enteropathy will likely influence the nature and amount of zinc delivered into the large intestine. Consequently, we reviewed the current literature to determine if colonic absorption of endogenous or exogenous (dietary) zinc could contribute to overall zinc nutriture. Whilst we found evidence that significant zinc absorption occurs in the rodent colon, and is favoured when microbially-fermentable carbohydrates (specifically resistant starch) are consumed, it is unclear whether this process occur in humans and/or to what degree. Constraints in study design in the few available studies may well have masked a possible colonic contribution to zinc nutrition. Furthermore these few available human studies have failed to include the actual target population that would benefit, namely infants affected by EE where zinc delivery to the colon may be increased and who are also at risk of zinc deficiency. In conducting this review we have not been able to confirm a colonic contribution to zinc absorption in humans. However, given the observations in rodents and that feeding resistant starch to children is feasible, definitive studies utilising the dual stable isotope method in children with EE should be undertaken.

## 1. Introduction

It has been estimated that nearly one third of the world’s population are receiving insufficient zinc from their diet [1]. In many low income countries, intake of animal products, the major source of dietary zinc in Western countries, is very low ([2]). Further compounding this is the fact that bioavailability of zinc from vegetarian diets, predominantly based on cereals and legumes, is affected by phytic acid—an absorption inhibitor due to its avid affinity for zinc [2]. Due to their high demand for zinc and limitations in body stores, children are particularly vulnerable to the consequences of zinc deficiency. Restricted growth, immune dysfunction, diarrhoea, and an increase in mortality can all be associated with clinical zinc deficiency in children [3]. Given the relevance of zinc deficiency to global health, considerable effort has been devoted to better understanding zinc nutrition, including zinc homeostasis. Whilst reduced bioavailability of dietary zinc is important, environmental enteropathy, a near universal condition among inhabitants of developing countries, might also affect zinc homeostasis due to changes in small intestinal morphology and function [4].

One question to emerge from such conjecture is whether the large intestine could then provide an auxiliary role for zinc absorption. Although evidence for colonic absorption of calcium, another divalent cation, exists for humans [5], there is little data to suggest a similar capacity for zinc absorption in the colon. However, as will be discussed, ignoring the colon’s potential to participate in zinc homeostasis may be premature, particularly as several animal studies strongly suggest that colonic absorption of nutritionally significant amounts of zinc can occur [6,7].

Thus the purpose of this review is to re-evaluate the current literature regarding the potential role of the human colon in zinc nutrition and consider what contribution, if any, the colon could make to improving zinc nutrition. It will also highlight particular areas of controversy and suggest areas where further research is required. 

## 2. Intestinal Zinc Homeostasis

The gastrointestinal tract is crucial for the homeostatic control of zinc and involves a complex interplay of host (including disease states), dietary and other environmental factors [8]. Apart from inadequate dietary intake of bioavailable zinc, other significant contributors to zinc deficiency may include the excessive intestinal loss of endogenously secreted zinc or impairment in small intestinal absorptive function [9]. Both of these conditions can occur in children suffering from environmental (or tropical) enteropathy, a condition characterized by blunting of the villi, and T-cell infiltration of the small intestine, which afflicts many inhabitants of developing countries [10]. Maintaining adequate zinc for bodily functions is dependent on absorption of some proportion of exogenous (dietary) zinc and reabsorption of endogenous zinc secreted into the gastrointestinal lumen. The latter likely arises mainly from digestive enzymes and cell shedding [8]. Ineffective homeostatic mechanisms aimed at conserving zinc or augmenting its absorption is evidenced by the widespread prevalence of zinc deficiency, particularly in vulnerable populations, such as children on marginal diets in countries where environmental enteropathy is prevalent [11]. 

If the colon is to participate in the absorption of luminal zinc, certain criteria must be satisfied: zinc delivered to the colon must be present in biologically important quantities, and the colonocyte must have the capacity to absorb this zinc and transfer it to the portal circulation. The zinc status of the host and by inference the body’s demand for zinc, may be an additional factor influencing colonic zinc absorption due to its effect on zinc transporters [12]. Whilst it has been reported that the zinc status of the host does not affect zinc absorption, the studies which helped derive this conclusion did not manipulate conditions to favour a colonic contribution to zinc absorption [13]. 

Before examining the potential for the colon to contribute to zinc absorption, we will briefly review the present state of knowledge of zinc absorption in the more proximal segments of the gut since delivery of zinc into the colonic lumen is likely to be influenced by changes in absorption and secretion proximally.

## 3. Dietary (Exogenous) Zinc

It is generally agreed that the majority of dietary zinc is absorbed in the proximal small intestine, through a transcellular saturable carrier-mediated mechanism [14]. Using a triple lumen steady state perfusion technique in healthy volunteers, the entire small intestine has been demonstrated to absorb zinc [14]. Although the jejenum appears to have the highest rate of zinc absorption, the duodenum is first exposed to zinc during the postprandial period and is likely to also contribute to zinc absorption [14]. During the ingestion of a meal, it is unlikely that the concentration of zinc in the lumen reaches the level required to saturate the transporters involved in zinc uptake from the lumen [15]. 

Many compounds found in the diet may affect the bioavailability of exogenous zinc and phytic acid (PA) is thought to be the most important of these [16]. When the molar ratio of dietary PA:Zn is increased, zinc absorption in the small intestine is progressively reduced [16]. In such a situation it would be expected that a greater portion of PA-bound exogenous zinc would be delivered into the more distal gut lumen. 

### 3.1. Zinc Transporters 

Absorption involves influx into the enterocyte across the apical membrane and then across the basolateral membrane and into the portal circulation [17]. As mentioned, zinc absorption, at least from the small intestine, is predominantly carrier-mediated and thus saturable [14]. There are two major families of zinc transporters: ZIP and ZnT [18]. The ZIP family of transporters regulates the influx of zinc into the cytosol, either from the lumen, serum or intracellular compartments. The ZnT family of transporters mobilize zinc in the opposite direction,* i.e.*, they facilitate zinc efflux from the cytosol to the extracellular environment or into intracellular organelles [18]. Within each family, multiple subfamilies exist and their abundance varies between organs. 

Both ZIP and ZnT transporters are expressed along the entire length of the gastrointestinal tract including the colon, raising the possibility of zinc absorption at more distal sites,* i.e.*, colon, provided that the luminal zinc is bioavailable and absorption is not suppressed by homeostatic control mechanisms [17]. 

Uptake from the intestinal lumen into the enterocyte is at least in part mediated by ZIP4, a protein located on the apical membrane of enterocytes [18]. In the mouse small intestine, experimental dietary zinc deficiency results in upregulation of ZIP4 mRNA and protein, accompanied by localisation of the ZIP4 protein to the enterocyte apical plasma membrane [19]. This homeostatic regulation might serve to increase zinc uptake from the lumen during periods of inadequate zinc intake [20], although whether this will be sufficient to maintain zinc nutrition is uncertain. 

We do not have parallel information in humans with zinc deficiency but it has been shown that an increase in dietary zinc intake can down-regulate expression of zinc transporters, including ZIP4 mRNA expression in the human small intestine [21]. The effects of reduced or increased dietary zinc on ZIP4 expression in the colon have not been studied in either experimental animals or humans. 

Mutations in ZIP4 are associated with acrodermatitis enteropathica (ADE), an autosomal recessive disorder characterised by dermatitis, alopecia and diarrhoea [22]. While this loss of ZIP4 function reduces uptake of dietary zinc from the lumen, oral administration of pharmacological quantities of zinc can correct zinc deficiency in affected patients with ADE [22], suggesting that other mechanism(s) of zinc absorption exist in addition to that mediated by ZIP4. 

## 4. Kinetics of Colonic Zinc Absorption

Although the transporters involved in zinc absorption, particularly ZIP4, are also expressed in the colon [23], it is unresolved as to whether absorption of zinc from the mammalian colon is predominantly transporter mediated or whether passive diffusion may also occur [24,25]. Although the tight junctions between the epithelial cells of the large intestinal epithelium are much tighter than those of the small intestine, this does not preclude the possibility of passive zinc absorption [26]. 

Using* in vitro* techniques which allow measurement of the unidirectional influx of zinc across the intestinal membrane of the rat, Condomina *et al.* demonstrated that zinc uptake from the colon increased linearly with the luminal concentration, suggesting that colonic zinc transport occurs via non-saturable diffusion [25]. In the human small intestine, the rate of zinc absorption saturates above a luminal concentration of 1.8 mM [14]. Condomina *et al.* demonstrated that zinc transport from the colonic lumen of the rat increases linearly above this concentration, with non-saturable diffusion demonstrated at luminal concentrations as high as 11.08 mM [25]. However, others have suggested that absorption of zinc from the rat colon exhibits combined kinetics,* i.e.*, both saturable and non-saturable components [24]. 

The kinetics of zinc absorption in the human large intestine has not been studied and it is difficult to reconcile the disparate findings in rodents. Differences in experimental techniques, diets, zinc status and strains of animals may account for some of the divergent observations. If the human colon were to be capable of non-saturable absorption of zinc, this might contribute significantly especially if zinc delivery to the colon is increased due to malabsorption or increased endogenous losses. More research is needed to prove or disprove such a theory. 

## 5. Colonic Absorption of Zinc in Rodent Models

Although colonic zinc absorption has been demonstrated* in vitro*, using rodent large intestine, it is not known whether colonic zinc absorption is of physiological significance (*i.e.*, quantitatively relevant), especially in the human. Some investigators have concluded that the overall contribution of zinc absorption from the caecum and colon is small [27,28]. For instance, Sorenson using an* in vivo* mouse model, quantified whole body and intestinal segment uptake of zinc using gamma counting of dietary 65Zn and concluded that there was little evidence to support colonic zinc absorption in zinc-replete animals fed a standard rodent diet [28]. 

However, using different experimental approaches, others have reached an alternative conclusion suggesting that physiologically significant absorption of zinc can indeed occur from the rat colon [6,7,29]. Hara *et al.* reduced proximal zinc absorption in rodents using omeprazole to increase luminal pH and found that serum zinc was lower in those rats which underwent caecocolectomy, compared to sham-operated rats [6]. They concluded that the colon may compensate for impaired zinc absorption in more proximal regions.

## 6. Promotion of Zinc Absorption by Microbially-Fermentable Substrates

The effects of altering colonic luminal conditions on zinc absorption in animals, supports the colon’s potential to manifest clinically significant zinc absorption [7,29,30].

Fermentable substrates are dietary carbohydrates that have resisted digestion and absorption in the upper gastrointestinal tract and upon entering the microbially-rich lumen of the colon, become available for fermentation [31]. Substrates such as dietary fibre, resistant starch, fructans and pentosans are fermented by resident colonic bacteria to produce short chain fatty acids (SCFAs),* i.e.*, acetate, propionate and butyrate [31]. As an energy source for colonocytes, SCFAs increase colonocyte cellular differentiation and mucosal cell proliferation [32]. They can also increase caecal blood flow and increase cation solubility by lowering luminal pH [32]. It has already been demonstrated that feeding fermentable substrates to rats enhances calcium and magnesium retention from the colon [33].

Several studies in experimental animals demonstrate the beneficial effect of the colonic fermentation of indigestible substrates on the absorption of zinc [7,29,30]. Several of the studies were conducted in animals fed diets containing a high PA: zinc molar ratio, limiting small intestinal absorption and thus ensuring a substantial delivery of zinc to the colon. Hayashi *et al.* also demonstrated that certain fibres could improve the growth rate in rats fed a high PA: zinc diet [34]. Such findings do not clarify the mechanism or kinetics of zinc absorption from the colon, but provide evidence that interventions known to affect colonic luminal conditions can increase zinc retention. 

## 7. Relevance of Zinc Status to Colonic Zinc Absorption

Although a reduction in dietary zinc has been shown to markedly increase fractional zinc absorption in humans (FZA), some experts assert that the zinc status of the host does not affect total zinc absorption [13]. Such studies, however, measured total intestinal zinc absorption, and have not been undertaken in a manner that exposes the host to prior factors that could increase the colon’s contribution to zinc absorption, whilst perhaps minimising the small intestine’s role in such absorption. Combining such conditions with manipulation of zinc status may confirm whether colonic absorption of zinc is subject to regulation by zinc status. It cannot be assumed that mechanisms and regulation of colonic zinc absorption are identical to those in the small intestine. In mice the change in level of expression of zinc transporters in response to zinc deficiency was higher in the colon than in the small intestine [20]. It was also shown in mice that mRNA expression of metallothionein, an intracellular protein that may influence zinc absorption by buffering intracellular zinc, was downregulated almost seven-fold in the colon but only 1.4-fold in the small intestine [20]. If however, there is significant passive absorption of zinc in the colon, independent of specific zinc transporters, then high concentrations of bioavailable zinc in the colonic lumen might facilitate absorption of zinc from the colon. Of course, in the absence of evidence to either support or refute such an observation, it remains only speculative. 

## 8. Phytase-Mediated Phytate Hydrolysis

Carbohydrates and their fermentation products provide a rich energy source for resident bacteria in the colon, some of which produce phytase, an enzyme capable of hydrolysing PA to phosphate and inositol phosphates [35]. The human gastrointestinal tract has very limited endogenous phytase activity [36]. The intermediate breakdown products of PA (inositol-6-phosphate), are more soluble and bind to minerals less avidly [15]. In a poultry model, it has been shown that feeding microbial phytases achieves luminal hydrolysis of phytate and facilitates increased retention of Ca, P, Mg and Zn by presumably increasing their bioavailability [37].

Bacteria are not the only source of luminal phytase as some plant foods also contain phytase [38]. Unfortunately, while dietary phytase is active in the upper gastrointestinal tract, especially in the acidic gastric environment, they are inactivated in the more alkaline pH of the small intestine [36]. Thus, microbial phytases might play a greater role, and it has been suggested that a potential mechanism by which fermentable substrates may improve zinc bioavailability and hence absorption is by promotion of phytase-mediated PA breakdown in the colon [7]. 

## 9. Endogenous Zinc and the Colon 

Under normal physiological circumstances, the primary site of absorption of exogenous (dietary) zinc in the human is thought to be in the proximal small bowel [8]. Zinc is also delivered to the lumen from endogenous sources, primarily from pancreatic (zinc-enzymes and metallothionien) and biliary secretions, sloughed cells, mucus and mucosal zinc secretion [39]. Thus a portion of endogenous zinc may require absorption at sites more distal to dietary zinc. The chemical form of endogenous zinc will also differ from that of dietary zinc and some of this endogenous zinc may also be trapped within an insoluble organic matrix, such as within shed epithelial cells. 

The amount of endogenous zinc in the lumen can be considerable [8]. Certain disease conditions are likely to impact on the magnitude of endogenous zinc and the amount of such zinc entering into the colon. More than 10^11^ enterocytes may be shed into the lumen on a daily basis, a number that may greatly increase during periods of infection or in disease such as environmental enteropathy [40]. Given that each eukaryotic cell has at least 200 µmoles of zinc, the daily amount of endogenous zinc entering the gastrointestinal lumen via these shed cells alone may amount to several milligrams [40,41]. Bacteria present in the large intestine are capable of digesting sloughed epithelial cells [42]. The fate of the intracellular zinc released from such digestion is unknown. If intake of bioavailable dietary zinc is low or there is active small intestinal disease reducing proximal zinc absorption, conservation of this endogenous zinc by the colon could be of particular importance to maintenance of adequate zinc nutrition. 

## 10. Zinc Absorption in Human Colon

There is a scarcity of human data to corroborate the findings in animals that the large intestine can participate in overall zinc absorption. The human colon certainly absorbs calcium [5]. Zinc absorption has been investigated in only a small number of subjects with extensive small bowel resection, and it is not possible to confidently deduce a role for the colon in zinc “salvage” from these results [43]. A single study found that absorption of zinc in patients following small bowel resection was similar to that in healthy subjects but as the purpose of the study was not to look at a colonic contribution to zinc absorption, the degree to which the colon remained in continuity varied and adaptation in the remaining small bowel might have been adequate to maintain overall zinc absorption [43].

Sandstrom, in a separate study instilled a radiolabelled zinc solution directly into the colon of healthy volunteers and measured whole body retention of the radionuclide. These investigators concluded that the human colon did not have the capacity to absorb clinically significant zinc [44]. More recently, it was found, by measuring retention of orally administered stable zinc isotopes, that there was no beneficial effect of a fructooligosaccharide on the absorption of zinc in postmenopausal women [45]. Similarly a study in young healthy male volunteers given inulin over a 28 day period found no beneficial effect on zinc absorption or balance [46]. Although these human studies offer no support for biologically significant zinc absorption from the human colon, there are several pertinent points that must be made, apart from the fact that only a very limited number of subjects were studied. In Sandstrom’s study the bowel preparation and hence clearing of luminal content including SCFAs might have affected the results [44]. The human studies undertaken so far did not explore the effect of zinc status of the participants and/or failed to create conditions that could maximise delivery of dietary zinc to the colon. The animal studies certainly suggest that colonic zinc absorption is likely to be of greater significance when small bowel absorption of zinc is somehow impaired [6,7]. It must also be considered that major differences exist between rodents and humans in lifespan, body proportion, enteric microflora composition and intestinal morphology, which may be responsible for these conflicting observations [47]. Systematic consideration of the multiple variables that might influence colonic zinc absorption and incorporation of these possible factors into the study design of future human studies will help resolve these issues.

## 11. Future Studies of Colonic Zinc Absorption

Several studies have investigated the importance of the colon in the absorption of other divalent cations, such as calcium. In a study of 118 patients with small bowel resections of various lengths, of which 38 had an ileostomy and 80 had part or whole of the colon remaining, Hylander *et al.* found that in patients with extensive small bowel resection the absorption of ^47^Ca was significantly higher when the colon was preserved [5]. There exists no study with similar methodology and with such a large number of participants, in the zinc literature. High doses of oral zinc have been demonstrated to overcome the absorptive defect in patients with acrodermatitis enteropathica (ADE), but there is no information whether the site of absorption of oral zinc in patients with ADE is the small intestine and/or the colon. Thus, will oral zinc correct zinc deficiency in a patient with ADE who has an ileostomy? 

Colonic perfusion studies would be informative for zinc absorption if they could be done while maintaining the usual luminal environment (*i.e.*, non-fasting and without bowel wash-out) but this might not be feasible especially in children. The effect of varying phytate intake would also be of interest. Certainly the dual isotope urine enrichment method has been extensively used to study zinc absorption in adult humans. Although, it does not distinguish between sites of absorption along the gut, it is still capable of capturing the colon’s contribution to this absorption. Thus one could utilise this well-validated method to determine if factors that could promote increased bioavailability of zinc from the colon could improve overall zinc absorption. Such a study design could involve feeding children with EE, two diets with either high or low fermentative capacity, both with equal high PA:Zn ratios, and then measure zinc absorption using the dual isotope enrichment method once a steady state in fermentation was achieved. The reasoning for the high PA:Zn ratio would be to mimic the usual diet of these children and minimise absorption of zinc in the small intestine due to compromised bioavailability. It must be ensured that both groups receive equal amounts of zinc in the diet and that this amount of zinc is sufficient to increase caecal zinc pool size. 

## 12. Luminal Conditions in the Large and Small Intestine

It must be remembered that the luminal conditions of the large intestine differ markedly from the small intestine. The composition and thickness of the mucus layer within the large intestine is likely to be different to that found in the proximal gut and subsequent interaction of minerals with the epithelial cell wall is also likely to differ. The transit time in the colon is much longer than for the proximal gut, potentially facilitating a prolonged interaction of bioavailable minerals (such as zinc) with its apical membrane transport system. The presence of potential ligands such as SCFAs, the concentrations of coexisting cations and the activity of microbial derived phytases may also influence mineral absorption. 

It is also possible that fermentative digestion of host proteins by the gut microbiota could liberate endogenous zinc from zinc-protein complexes since it is known that fermentation of dietary proteins occurs [48]. But the degree to which bacteria resident in the gut may sequester this zinc for their use or make it available for absorption is not known [49]. Alterations in the microbiota by the provision of a greater or lesser quantity of luminal zinc in the colon, and the clinical consequences have not been adequately studied. Furthermore children with environmental enteropathy have a gut dysbiosis, and how this may affect the release of zinc from phytate or endogenous proteins is also unknown [49]. 

Ultimately the amount of zinc that enters the colon is dependent on the quantity of exogenous and endogenous zinc delivered into the lumen and the homeostatic control of their absorption and secretion proximal to the colon. Although the use of stable zinc isotopes can enable separate examination of net faecal excretion of endogenous and exogenous zinc, studies employing this technique were not designed to discriminate between the possible role of the colon in endogenous and exogenous zinc absorption, particularly in conditions that could facilitate colonic zinc absorption.

## 13. Conclusions 

This review concludes that significant zinc absorption does occur in the rodent colon, particularly when fermentable substrates are fed. Whether it is influenced by whole body zinc status remains to be determined. In acknowledging the considerable limitations in the human studies to date, it remains unresolved as to whether feeding fermentable substrates could improve zinc bioavailability in those who are at risk of zinc deficiency, have impaired small intestinal function and/or experience substantial endogenous losses. These are the very conditions faced by many children in low-income countries. The few available human studies which have led to general dismissal of the colon’s capacity to participate in zinc absorption, have not included the actual target population that we would be interested in pursuing a novel zinc intervention, namely infants affected by EE where zinc delivery to the colon may be increased, perhaps on a background of preceding zinc deficiency. In conducting this review we have not been able to confirm colonic absorption of zinc in humans. However, given the observations in rodents and that feeding resistant starch to children is feasible, definitive studies utilising the powerful dual stable isotope method in children with EE should be undertaken.

Globally, zinc deficiency remains a major concern despite decades of research highlighting its negative effects on human and, in particular, child health. One DALY (disability-adjusted life year) equates to approximately 1 lost year of “healthy life”, and it has been estimated that in one year alone the burden of zinc deficiency may equate to over 16 million DALYs and over 450,000 deaths in children under 5 years of age [50]. Given the magnitude of this issue, examining the potential for the human large intestine to contribute to zinc nutrition warrants such discussion, particularly as it is a possibility that has largely been dismissed. It also requires envisaging a definitive experiment to prove or disprove such a theory. Finally, evidence of apparent absorption of other divalent cations, such as calcium and magnesium by the rat colon appears to correspond with eventual demonstrable absorption of these same minerals in the human large intestine [33,51,52]. The potential failure of zinc to follow such a pattern may itself be of some interest.
